# The Babyccino: The Role of Caffeine in the Prevention of Acute Kidney Injury in Neonates—A Literature Review

**DOI:** 10.3390/healthcare12050529

**Published:** 2024-02-23

**Authors:** Nimisha Aithal, Yogavijayan Kandasamy

**Affiliations:** 1 Department of Pediatrics, Townsville University Hospital, Townsville, QLD 4811, Australia; 2 Department of Neonatology, Townsville University Hospital, Townsville, QLD 4811, Australia; 3 College of Medicine and Dentistry, James Cook University, Townsville, QLD 4814, Australia

**Keywords:** caffeine, caffeine citrate, acute kidney injury (AKI), neonate, premature

## Abstract

Acute kidney injury (AKI) in neonates is associated with increased morbidity and mortality. Theophylline (a methylxanthine) has been shown to prevent neonatal AKI but is seldom used due to its unfavorable profile. Caffeine, another methylxanthine, is utilized ubiquitously to treat apnea of prematurity, but there are no randomized trials evaluating its efficacy in preventing neonatal AKI. This literature review aims to summarize the existing research pertaining to the relationship between caffeine and neonatal AKI. The review was conducted using Pubmed, Embase, Google Scholar, and Cochrane. Inclusion criteria incorporated empirical studies, being published in English, and being available electronically. All eight studies identified were included. Seven studies found caffeine-exposed premature neonates had lower rates of AKI than caffeine-unexposed neonates. Four found reduced AKI severity with caffeine exposure. One study included term neonates and did not find a difference in the AKI rate between caffeine-exposed and non-exposed babies. Limitations include exclusively observational studies, short study periods, heterogenous definitions of prematurity, and a lack of assessment of dose–effect relationships. In conclusion, premature neonates exposed to caffeine appear to have lower rates and potentially less severe AKI. Further research is needed before caffeine can be considered for use in the primary prevention of neonatal AKI.

## 1. Introduction

Approximately 1 in 10 babies are born prematurely (<37 weeks gestational age (GA)), equating to an estimated 15.2 million pre-term births each year worldwide [1,2,3]. Globally, complications associated with prematurity are the leading cause of childhood morbidity and mortality with preterm birth accounting for 36.1% of neonatal deaths and 17.7% of deaths in children <5 years of age [3,4]. Within the neonatal period, one such complication is acute kidney injury (AKI) which occurs in 30% of all neonates, with almost 50% of those born <29 weeks GA affected [5].

Neonatal AKI is diagnosed and classified using the Kidney Disease Improving Global Outcomes (KDIGO) classification modified for newborns [6]. This classification encompasses increased serum creatinine (sCr) values in a tiered fashion over the baseline as well as a reduction in urine output (UO) over time [6]. Stage 1 AKI is defined as sCr rise by ≥0.3 mg/dL within 48 h, 150–190% of the trough value within 7 days or UO of >0.5 and ≤1 mL/kg/h over 24 h. Stage 2 is defined as sCr rise ≥ 200–290% of trough value or UO >0.3 and ≤0.5 mL/kg/h. Stage 3 is defined as sCr ≥ 2.5 mg/dL, sCr rise ≥ 300% of the trough value, UO ≤ 0.3 mL/kg/h or receipt of dialysis [6].

The Assessment of Worldwide Acute Kidney Epidemiology in Neonates (AWAKEN) study was an international multicenter retrospective observational cohort study conducted across 24 NICUs to ascertain the relationship between AKI and neonatal morbidity and mortality [5]. It found that AKI was associated with higher mortality (10% vs. 1%; *p* < 0.0001) and a longer hospital stay (median 23 days vs. 19 days; *p* < 0.0001) than those without AKI. This distinction persisted after the adjustment of factors including gestational age, indication for admission, mode of delivery, need for resuscitation, APGAR score at 5 min, maternal anti-inflammatory use, and neonatal growth parameters. Thus, as neonatal AKI is common and associated with adverse outcomes, the prevention and management of it is paramount in the care of babies.

To date, theophylline (a methylxanthine) is the only medication shown to prevent neonatal AKI in randomized control trials, specifically in neonates with severe birth asphyxia [7,8,9,10,11,12,13]. Whilst effective for AKI prevention and treatment of apnea of prematurity (AOP), theophylline is not widely used due to its narrow therapeutic index, need for frequent assessment of serum drug levels, short half-life, and potential for cardiovascular side effects [14,15,16]. Caffeine, another methylxanthine, is used ubiquitously in neonatal units, and, in light of this, there is increasing interest in the effect of caffeine on neonatal kidneys and the role it could play in neonatal renal outcomes. 

Caffeine was first used to treat AOP in 1977, but it was not until 2006 when The Caffeine Therapy for Apnea of Prematurity (CAP) trial confirmed that it was superior to the placebo [17,18]. Since then, multiple studies and systematic reviews have not only shown that caffeine is effective in the treatment of AOP, but that it can also facilitate successful extubation, shorten the duration of positive pressure ventilation, reduce rates of bronchopulmonary dysplasia (BPD), lessen intervention for patent ductus arteriosus, and may improve neurodevelopmental outcomes [17,19,20,21,22,23,24,25,26,27]. Currently, caffeine is one of the most used drugs in neonatology, it is cost-effective with treated neonates having simultaneously better outcomes and lower mean costs, and multiple national and international bodies recommend it as a first line in the treatment of AOP and to improve the success of extubation [28,29,30,31,32,33]. 

Caffeine citrate can be administered intravenously (IV) or orally [14]. It is rapidly absorbed after enteral administration, has complete bioavailability due to minimal first pass metabolism, and does not require dose adjustment when switching between routes [14,19,34]. In neonates, ~86% of caffeine citrate is excreted unchanged in the urine, and the remainder is hepatically metabolized via the CYP1A2 enzyme system [35]. The plasma half-life of caffeine reduces with advancing age as the renal function of neonates matures with time, and the maturity of the hepatic enzymes is dependent on post-natal age regardless of birthweight or gestational age at birth [36]. Standard caffeine dosing of a 20 mg/kg load followed by 5–10 mg/kg once daily has been shown to result in adequate therapeutic levels in 70% of neonates [37]. Routine therapeutic drug monitoring is not required because toxic serum levels rarely occur within the recommended dosing range [38,39]. 

Whilst caffeine is a safe drug, it is associated with side effects including tachycardia, hypertension, tremors, and gastroesophageal reflux [14,40]. Caffeine has been shown to increase oxygen consumption and energy expenditure leading to slow weight gain [41]. The CAP trial found that caffeine-treated neonates gained less weight than their non-caffeine-exposed counterparts in the first 2 weeks of treatment (mean difference 23 g, *p* < 0.001), but this did not correlate with a long-term difference in weight gain at an 18- and 21-month follow up [17]. In toxic doses, tonic–clonic movements, opisthotonos, hyperglycemia, and metabolic acidosis have been reported [42]. These are rare and abate with reduction or cessation of caffeine [34,42].

At therapeutic doses, caffeine is an adenosine receptor antagonist acting primarily at A1 and A2a receptors [40]. At supratherapeutic concentrations, caffeine acts as a phosphodiesterase inhibitor and increases active intracellular calcium mobilization [40]. Caffeine decreases AOP primarily through stimulation of the medullary respiratory centers and increases their sensitivity to carbon dioxide and hypoxia. It also increases diaphragmatic activity, minute ventilation, and pulmonary blood flow [43,44]. 

In the kidneys of preterm neonates, caffeine is shown to increase the glomerular filtration rate (GFR), creatine clearance, urine calcium excretion, UO, and induce diuresis [45,46]. However, the precise mechanism by which caffeine elicits these reno-protective effects is not fully known. It has been suggested that the A1 receptor antagonism decreases afferent arteriolar pressure thus increasing renal blood flow and GFR [47]. However, a study of caffeine in newborn rabbits found that whilst caffeine was associated with a delayed increase in renovascular resistance and filtration fraction, it did not significantly alter renal blood flow [48]. There is a paucity of research into caffeine’s effect in renal protection during hypoxia. A 2021 study of 31 neonates <32 weeks of gestational age found that caffeine increased renal regional saturation of oxygen (RSO2) in the first week in those with low RSO2 but had no effect in those with normal RSO2 [49]. However, a study in neonatal rats showed that high caffeine doses in those exposed to intermittent hypoxia may be associated with histopathologic evidence of AKI [50,51]. Ultimately, as available studies have small sample sizes or are conducted in animals using doses and serum caffeine targets different to those used in clinical practice, more research is needed to better understand the precise mechanism of action of caffeine in the human neonatal kidney.

Nevertheless, considering that caffeine is commonly used in the preterm demographic and is in the same drug class as theophylline, the purpose of this literature review is to explore the existing evidence on the use of caffeine in neonatal AKI prevention to determine if it is a potential option in preventing AKI and thus reducing neonatal morbidity and mortality.

## 2. Materials and Methods

The authors separately conducted comprehensive literature searches on key medical and health databases including Pubmed, EMBASE, Google scholar, and Cochrane Central Register of Controlled Trials. Studies relating to or examining the relationship between caffeine use and neonatal AKI were sought from database inception to 30th November 2023. Several keywords and their combinations were used to retrieve articles from these databases, including “neonate”, “premature”, “infant”, “acute kidney injury”, “AKI”, “renal insufficiency”, “caffeine”, “caffeine citrate”, and “methylxanthine”. 

Inclusion criteria incorporated empirical studies, being published in English, and being available electronically. Studies conducted on animals were excluded. Reference lists of the retrieved articles were manually screened. Studies were selected based on their title, abstract, and method. No automation tools were utilized. The authors identified the same publications, and all are included in this review.

## 3. Results

The exploratory search found eight primary studies which are summarized in Table 1. In this section, each included study is described and their key findings are presented. 

In the largest study looking at the relationship between caffeine and neonatal AKI, Harer et al. carried out a secondary analysis of 675 neonates included in the AWAKEN study to specifically examine the association between caffeine and AKI in the first 7 days of life in neonates <33 weeks GA [53]. They found that caffeine administration in the first 7 days was associated with less-frequent AKI compared to no caffeine exposure (11.2% vs. 31.6%, *p* < 0.01). This benefit persisted after multivariable adjustment, with caffeine-receiving neonates having a reduced odds of developing AKI (adjusted odds ratio (OR) 0.20; 95% CI, 0.11–0.34). Additionally, their analysis found that those receiving caffeine were less likely to develop more severe (stage 2 or 3) AKI with an adjusted OR of 0.20 (95% CI, 0.12–0.34). The number needed to treat (NNT) was 4.3. It was noted that caffeine-exposed neonates were more likely to be younger, sicker, and ventilated. As the caffeine dose was not collected in the AWAKEN study, the dose-dependent effect could not be studied. 

Four similarly constructed prospective cohort studies have been conducted in neonates of various prematurity to ascertain differences in AKI rates between caffeine- and non-caffeine-exposed babies [55,56,57,58]. All studies included 100 neonates each and found that neonates given caffeine had lower prevalences of AKI. All four also noted lower peak SCr levels in caffeine-exposed neonates; three reached statistical significance [56,57,58].

Looking at neonates <30 weeks GA (using New Ballard score), Mansour et al. examined the relationship between caffeine administration within the first 24 h of life and the likelihood of developing AKI within the first 7 days [56]. All caffeine-exposed infants received 20 mg/kg of IV caffeine citrate as a loading dose, followed by 5 mg/kg every 24 h for 1 week. They found that there was no difference in the baseline serum creatinine between those who did and did not receive caffeine, but that caffeine-exposed neonates had lower serum creatinine at day 5 (0.67 ± 0.25 mg/dL) and 7 (0.78 ± 0.49 mg/dL) of life compared to those not given caffeine (0.87 ± 0.18 mg/dL and 1.20 ± 0.30 mg/dL, *p* < 0.001). Although they reported less AKI in the caffeine-exposed group (20% vs. 54%), the criteria for AKI was not provided. Notably, there was no statistically significant difference between caffeine- and non-caffeine-exposed neonates in terms of a need for invasive ventilation, their mean length of stay, or mortality. 

In a slightly older cohort of neonates, Mohamed et al. examined the association between early caffeine citrate administration and the risk of acute kidney injury in hospitalized neonates born at 32–35 weeks GA [57]. They found that, in caffeine-exposed neonates, sCr was lower at day 2 (0.81 mg/dL vs. 1.08 mg/dL, *p* < 0.001) and day 7 (0.53 mg/dL vs. 0.85 mg/dL, *p* < 0.001) compared to the non-exposed group. They also found that there was a significantly higher occurrence of apnea in neonates who did not receive caffeine compared to those who did (80% vs. 20%, *p* < 0.001). Information about the caffeine dose was not provided.

Sivasaranappa et al. and Al Hamshary et al. have carried out prospective studies in neonates <37 weeks GA [55,58]. Sivasaranappa et al. examined the association between caffeine administration and risk and severity of AKI occurring in the first 7 days after birth [58]. They found that those who received caffeine were less likely to develop early AKI compared to those who did not (17.5% vs. 44.2%, *p* = 0.004) and that caffeine-exposed neonates had less-severe AKI than their non-exposed counterparts [(Stage 2 AKI 8.8% vs. 20.9%, *p* = 0.02) and (Stage 3 AKI 1.8% vs. 11.6%, *p* = 0.02)]. Notably, neonates receiving caffeine were more likely to have an earlier GA, lower birthweight, lower APGAR, needed resuscitation at birth, and received mechanical ventilation. Al Hamshary et al. included neonates <37 weeks GA who also required IV fluids for at least 48 h [55]. They found that caffeine-exposed infants had lower rates of AKI (38% caffeine group vs. 52% no-caffeine group, *p* = 0.015). There was also a lower rate of stage 1 AKI (8% vs. 30%, *p* = 0.011), but not of stage 2 (0% vs. 6%, *p* = 0.041) AKI as defined by the study *p* value criteria (noting that the *p* value for the latter was <0.05). Similar to Sivasaranappa et al.’s findings, they also found that those given caffeine were more likely to be more premature and have lower birth weights. Neither study collected data on caffeine dose, and, thus, the dose–effect correlation could not be ascertained. 

The association between caffeine exposure and AKI within the first 10 days of life in very-low-birth-weight (VLBW) (≤1500 g) neonates and in those undergoing prolonged invasive ventilation was explored by Carmody et al. in a retrospective cohort study of 140 VLBW patients [52]. They found that AKI occurred less frequently in neonates who received caffeine (17.8% versus 43.6%, *p* = 0.002) and that caffeine was associated with decreased odds (OR 0.22; 95% CI 0.07–0.75, *p* = 0.02) for AKI in logistic regression models adjusted for other variables. Although neonates exposed to caffeine were less likely to develop stage 1 AKI, there was no difference in the frequency of stage 2 and 3 AKI compared to caffeine-unexposed neonates. In a sub-group analysis of neonates undergoing prolonged ventilation, AKI occurred less frequently in neonates treated with caffeine (29.2% with caffeine, vs. 75.0% without caffeine, *p* = 0.002); however, again, there was no difference in the occurrence of severe (stage 2 or 3) AKI. Mean GA in caffeine-exposed and non-exposed neonates was 27.3 and 28.4 weeks, respectively. Caffeine exposure was defined as a 20 mg/kg loading dose and 5 mg/kg/day maintenance dose, with the option to increase the dose at the clinician’s discretion. NNT was 2.87 across all neonates and 5.02 in neonates requiring prolonged invasive respiratory support. As only a small minority of patients had a caffeine level measured; the dose–effect relationship could not be ascertained.

Aviles-Otero et al. sought to examine the association between caffeine exposure and AKI in a cohort of 146 neonates <34 weeks GA with NEC and/or SIP and found that the renal benefit of caffeine may extend into this sub-group of neonates [54]. In their study, AKI was less frequent in those receiving caffeine (55.5% vs. 92.6%) with an OR of AKI with caffeine of 0.1 (95% CI, 0.02–0.44). This association persisted in a multivariable analysis (adjusted OR 0.08, CI 0.01- 0.42). The NNT was 2.6. When looking at serum creatinine levels, those who had received caffeine had lower peak creatinine (median 1.0 mg/dL vs. 1.5 mg/dL; *p* = 0.008) and absolute creatinine change (median 0.42 mg/dL vs. 0.68 mg/dL; *p* = 0.003). There was no difference in baseline serum creatinine. Caffeine exposure in this study was defined as a loading dose (40 mg/kg) followed by a daily maintenance dose (8 mg/kg). 

As caffeine is given primarily for apnea of prematurity, there is limited data regarding renal outcomes in neonates >37 weeks GA. The only study to date involving term neonates was conducted by Thompson et al. using population pharmacokinetic (PK) modelling techniques [59]. It included 132 neonates, all born at term, undergoing cardiac surgery requiring cardiopulmonary bypass (CBP) within the first 28 days of life to explore associations between caffeine exposure and cardiac-surgery-associated AKI (CS-AKI). Participant recruitment came from two prospective trials: the Steroids to Reduce Inflammation after Infant Heart Surgery (STRESS) trial, and the Opportunistic PK/Pharmacodynamic Trial in Critically Ill Children with Heart Disease (OPTIC). In total, 23 caffeine-exposed neonates and 109 matched controls were included. In the caffeine group, 85% (23/27) received a 20 mg/kg loading dose, and the median pre-operative dose was 5 mg/kg. On univariate analysis, caffeine-exposed neonates more frequently had single-ventricle heart disease (43% vs. 29%), underwent surgery earlier (age 3 vs. 7 days), had shorter CPB times (median 138 vs. 177 min), and were more frequently female (64% vs. 52%). Neonates who developed AKI were more likely to have single-ventricle heart disease (44% vs. 18%) and have longer CBP time. In a multivariable analysis, there was no significant difference in the rate of CS-AKI with or without caffeine exposure. Similarly, none of the simulated caffeine exposure parameters were associated with decreased odds of severe AKI. In a subgroup analysis of neonates with single-ventricle heart disease, there was no difference in CS-AKI rates depending on caffeine exposure. Whilst those without CS-AKI had higher caffeine exposures, this did not reach significance. In terms of analysis of pharmacokinetics, the majority of patients had subtherapeutic (for apnea of prematurity) serum caffeine levels. Caffeine-exposed neonates had an average drug clearance of 0.43 L/h/70 kg which is in keeping with existing studies in preterm neonates, but the volume of distribution was 25% higher in neonates undergoing CBP than in the literature’s values.

## 4. Discussion

The research to date evaluating the relationship between caffeine and AKI in premature neonates unanimously suggests that preterm babies receiving caffeine have lower rates of AKI [52,53,54,55,56,57,58]. This relationship has also been shown to persist after multivariable adjustment [52,53,54]. The number needed to treat them varies between studies from 2.6 to 5.02 [52,53,54]. NEC, SIP, and prolonged ventilation are all recognized complications of prematurity, and although the number of studies in these sub-populations of neonates is limited, it appears that they also experience renal benefit from caffeine exposure [52,54]. Data on term neonates is lacking with only one study having looked at neonates born >37 weeks GA [59]. This study specifically assessed the effect of caffeine on CS-AKI and found that caffeine exposure did not reduce rates of CS-AKI. More research is needed to ascertain if older gestation, type of cardiac co-morbidity, CBP time, or altered pharmacokinetics of caffeine in this specific population contributed to the observed results.

With regard to AKI severity, there is variation between studies when exploring the relationship between caffeine and serum creatine rise (a key marker used to grade AKI). Harer et al., Mansour et al., and Sivasranappa et al. found that neonates exposed to caffeine were also less likely to develop more severe AKI (stage 2 or stage 3) as defined by serum creatinine rise [53,56,58]. Aviles-Otero et al. found that neonates exposed to caffeine have lower peak serum creatinine levels and lower absolute creatinine change [54]. Contrary to these findings, two included studies found that, although caffeine-exposed neonates had lower rates of stage 1 AKI, there was no difference in stage 2 or stage 3 AKI compared to caffeine-naive neonates [52,55]. Further studies are required to ascertain if caffeine exposure leads to a reduction in more severe and subsequently clinically significant AKI, and if so, which sub-sets of neonates stand to benefit most. 

Data surrounding caffeine and long-term renal outcomes is scant. Harer et al. conducted the only study to date exploring long-term kidney implications of neonatal caffeine exposure by looking at 598 caffeine-exposed ex <28 week neonates at 22–26 months corrected age [60]. They found that neonatal caffeine exposure was not associated with any abnormal measures of kidney function at 2 years. In a subgroup analysis, caffeine-exposed children without BPD had a lower adjusted OR (aOR 0.78) of having eGFR of <90 mL/min/1.73 m^2^, whilst those with BPD had a higher adjusted OR (aOR 1.15) of elevated blood pressure. Notably, the 95% confidence interval for both analyses approached 1 (0.62–0.99 and 1.05–1.25, respectively), and the relationship between those suffering neonatal AKI and subsequent long-term renal outcome was not explored. Thus, at the present time, it is unclear if the reduction in AKI rates seen in caffeine-exposed neonates translates to long-term renal benefit. More research into neonatal caffeine exposure and longitudinal renal outcomes is needed. 

There are several limitations within the existing literature surrounding caffeine and AKI in neonates. All studies to date are observational, seven of the eight studies had small sample sizes (<200), and inclusion criterion varied between studies with the definition of prematurity ranging from <30 weeks gestational age to <37-weeks gestational age [52,53,54,55,56,57,58,59]. Only one study included term neonates [59]. Thus, larger scale prospective randomized clinical trials including neonates with comparable gestational ages (pre-term and term) are required to better evaluate the effect of caffeine on AKI prevention and treatment in neonates. As impaired nephrogenesis and increased risk of prematurity-related complications increases with earlier GA, this makes direct comparison of existing findings challenging, and illustrates a need to delineate the renal benefit of caffeine at various gestations to ascertain which neonates stand to benefit. The largest of the studies classified neonates who commenced caffeine after suffering AKI as “not exposed to caffeine”, whilst the remainder solely looked at the relationship between caffeine exposure and later development of AKI [53]. Thus, the effect of caffeine in neonates with established AKI, the time to AKI resolution, and if there is a benefit to commencing it in this sub-set of patients is an area for further research. Existing studies either did not report caffeine dosing, or used differing caffeine doses, and only one correlated outcome measures with serum caffeine levels. Consequently, the dosage–effect relationship between caffeine and AKI is not clear. Etiology of AKI was not established in any study, and thus a sub-group analysis by AKI type was not carried out. Only three studies investigated sub-groups of neonates with the comorbidities of NEC, SIP, prolonged ventilation, and cardiac surgery [52,54,59]. Thus, more research is needed to elucidate if the benefit of caffeine is seen in pre-renal, renal, or post renal AKI as well as to investigate which comorbid neonates are likely to benefit most from caffeine’s renal-protective effects. Seven of the eight studies utilized KDIGO criteria for AKI, which incorporate urine output and SCr as measures for AKI due to their practicality in clinical practice. However, it must be noted that neonates more frequently undergo non-oliguric renal failure, and serum creatine is shown to be an inaccurate measure of AKI due to its dependence on muscle mass, reabsorption in renal immaturity and interference of maternal sCR levels [61]. Additionally, the majority of studies used the Jaffe method for SCr measurement which can falsely elevate SCr due to non-specific protein interference (e.g., bilirubin) or falsely underestimated SCr at lower levels [62]. Given SCr measurements are low in neonates and hyperbilirubinemia is common, AKI may have been incorrectly estimated in the existing data and future research utilizing enzymatic methods for SCr measurement (which have been proven to be more accurate) or alternative markers (e.g., Cystatin C) should be considered [63,64]. 

## 5. Limitations

This review is narrative, not systematic. Thus, although a thorough literature search was conducted, there may be additional papers which are not included despite meeting inclusion criteria. Additionally, whilst the current available literature has been discussed, rigorous protocolized analysis into the quality and biases within the studies, and consequent adjustment in weight was not carried out. As this is a narrative review, the review was not registered.

## 6. Conclusions

Caffeine exposure is associated with lower rates of AKI in the acute period in premature, but not term, neonates. It is unclear whether caffeine exposure reduces AKI severity, length of stay, morbidity, or mortality. The optimum dose of caffeine required for neonatal renal protection is not known. Whilst caffeine use in the prevention of neonatal AKI is a promising prospect, further studies focusing on neonates of varied gestational ages and comorbidities, the long-term clinical outcomes, and the elucidation of caffeine dose–effect relationships are needed before caffeine can be realistically considered for use in the primary prevention of neonatal AKI. 

## Figures and Tables

**Table 1:** Comparison of studies to date investigating caffeine and neonatal AKI.

Author(s)	Year	Study Type	Aim(s)	Outcome/s	Number of Neonates	Inclusion GA (Weeks)	Mean GA of Caffeine-Exposed Neonates (Weeks)	Mean GA of Caffeine-Unexposed Neonates (Weeks)	Results
Carmody et al. [52].	2016	Retrospective cohort study	To evaluate the association between caffeine exposure and acute kidney injury (AKI) in very-low-birth-weight (≤1500 g) neonates.	Primary outcome: AKI within the first 10 days after birth according to the KDIGO system, modified to include only serum creatinine.	140	N/A	27.3	28.4	▪ Caffeine exposure was associated with lower AKI rate. ▪ Caffeine exposure was associated with less stage 1 but not stage 2 or stage 3 AKI. ▪ NNT (all neonates) was 2.87. ▪ NNT (neonates requiring prolonged ventilation) was 5.02.
Harer et al. [53].	2018	Secondary analysis of the AWAKEN study	(1) Examine the association between caffeine and AKI in preterm neonates in the first 7 days of life. (2) Test the hypothesis that caffeine is associated with reduced rate and severity of AKI.	Primary outcome: Incidence of AKI (based on the modified neonatal KDIGO definition) in the first 7 days after birth. Secondary outcomes: Severity of AKI, and incidence of AKI within the data collection period.	675	<33	28.5	29.6	▪ Caffeine exposure was associated with lower AKI rate. ▪ Caffeine exposure was associated with less-severe AKI (stage 2 and stage 3). ▪ NNT 4.3.
Aviles-Otero et al. [54].	2019	Retrospective cohort study	To evaluate the association between caffeine exposure and AKI in a cohort of patients with NEC and SIP.	Primary outcome: Incidence of AKI (based on the modified neonatal KDIGO definition) 24 h before or up to 1 week after diagnosis of NEC or SIP. Secondary outcome: Degree of sCR increase.	146	<34	26	25	▪ Caffeine exposure was associated with lower AKI rate. ▪ Caffeine exposure was associated with lower peak sCR and lower absolute change in sCR. ▪ NNT 2.6.
Al Hamshary et al. [55].	2021	Prospective cohort study	To determine if administering caffeine citrate to premature infants will alter the occurrences of AKI.	Primary outcome: Incidence of AKI (based on the modified neonatal KDIGO definition) at day 7. Secondary outcome: Severity of AKI.	100	<37	32.6	34.8	▪ Caffeine exposure was associated with lower AKI rate. ▪ Caffeine exposure was associated with less stage 1 but not stage 2 or stage 3 AKI.
Mansour et al. [56].	2021	Prospective cohort study	To determine if preterm infants administered caffeine in the first 24 h of life are less likely to develop AKI within the first 7 days.	Primary outcome: Incidence of AKI at 7 days of age. Secondary outcomes: sCR, serum urea, serum albumin, and urine output.	100	<30	28.5	28.5	▪ Caffeine exposure was associated with lower AKI rate. ▪ Caffeine-exposed neonates had lower day 5 sCr.
Mohamed et al. [57].	2021	Prospective cohort study	To determine association between early caffeine administration and risk of AKI in hospitalized preterm neonates.	Primary outcome: Incidence of AKI (based on the modified neonatal KDIGO definition) at 7 days of age. Secondary outcome: Incidence of apnea within 7 days of age.	100	32–35	Not provided	Not provided	▪ Caffeine exposure was associated with lower sCR at day 2 and day 7.
Sivasaranappa et al. [58].	2020	Prospective cohort study	To study the association between caffeine administration and risk and severity of early AKI occurring in the first 7 days after birth.	Primary outcome: Early AKI (based on the modified neonatal KDIGO definition) occurring in the first 7 days after birth. Secondary outcome: Severity of AKI.	100	<37	Not provided	Not provided	▪ Caffeine-exposed neonates were less likely to have AKI. ▪ Caffeine-exposed neonates had less severe AKI.
Thompson et al. [59].	2023	Mixed cohort study	(1) To address knowledge gaps in age-, disease-, and bypass-related effects on caffeine disposition. (2) Explore preliminary associations between caffeine exposure and CS-AKI using population pharmacokinetic modeling techniques and an opportunistic, electronic health record-integrated trial design.	Primary outcome: CS-AKI (based on the modified neonatal KDIGO definition).	132	N/A	39.3	39	▪ No significant difference in rate of CS-AKI with or without caffeine exposure.

Abbreviations: Gestational age (GA), acute kidney injury (AKI), Kidney Disease Improving Global Outcomes (KDIGO), number needed to treat (NNT), necrotizing enterocolitis (NEC), spontaneous intestinal perforation (SIP), serum creatinine level (sCR), cardiac-surgery-associated acute kidney injury (CS-AKI).

## Data Availability

Data are contained within the article.
